# Supplementary material for: Reliability and Validity of Computerized Adventitious Respiratory Sounds in People with Bronchiectasis
Source: J Clin Med. 2022 Dec 19;11(24):7509. doi: 10.3390/jcm11247509 (PMC9787476; doi:10.3390/jcm11247509)
Supplement: Supplementary file 1 [file jcm-11-07509-s001.zip › jcm-2073088-supplementary.pdf]

Supplementary material

Table S1. Descriptive characteristics of crackles recorded in both assessment sessions.

	Session 1						Session 2					
	Recording 1			Recording 2			Recording 3			Recording 4		
	All	Anterior	Posterior	All	Anterior	Posterior	All	Anterior	Posterior	All	Anterior	Posterior
Total number of crackles												
Inspiratory phase	1.2 [1.0-2.6]	1.2 [0.6-2.0]	1.5 [0.9-2.6]	1.4 [0.6-2.5]	1.2 [0.6-3.1]	1.4 [0.8-2.3]	1.3 [0.8-2.1]	1.0 [0.7-1.6]	1.8 [0.9-2.7]	1.2 [0.8-2.6]	1.1 [0.5-2.2]	1.3 [1.0-3.0]
Expiratory phase	2.3 [1.7-3.3]	2.4 [0.9-3.5]	2.5 [1.7-3.9]	2.0 [1.2-3.9]	2.0 [0.8-2.9]	2.0 [1.3-4.1]	1.7 [1.4-3.5]	1.6 [1.1-2.8]	1.9 [1.2-3.9]	2.0 [0.7-3.3]	1.7 [0.8-2.4]	2.0 [1.0-3.3]
Complete respiratory cycle	3.9 [2.6-5.1]	3.5 [1.8-5.6]	4.2 [2.7-5.2]	3.6 [1.9-6.4]	3.3 [1.5-5.7]	3.6 [2.3-6.6]	3.0 [2.2-5.7]	2.6 [1.9-4.2]	3.4 [2.7-6.5]	3.4 [1.6-6.1]	2.8 [1.3-4.9]	4.2 [2.2-6.2]

The values of crackles obtained in right and left side were pooled.

Table S2. Descriptive characteristics of wheezes recorded in both assessment sessions.

	Session 1						Session 2					
	Recording 1			Recording 2			Recording 3			Recording 4		
	All	Anterior	Posterior	All	Anterior	Posterior	All	Anterior	Posterior	All	Anterior	Posterior
Total number of wheezes												
Inspiratory phase	0.8 [0.5-2.5]	0.8 [0.4-2.2]	0.8 [0.3-2.0]	0.9 [0.7-2.2]	1.0 [0.4-2.0]	0.7 [0.5-1.4]	1.0 [0.2-1.7]	0.8 [0.1-1.7]	0.6 [0.3-1.8]	0.9 [0.2-2.4]	0.8 [0.1-2.4]	0.7 [0.2-2.2]
Expiratory phase	2.6 [1.5-4.5]	1.7 [1.2-2.9]	3.3 [1.7-5.5]	2.4 [1.6-4.3]	1.8 [1.2-3.7]	2.8 [1.6-4.7]	1.7 [0.9-4.1]	1.7 [0.5-5.5]	1.8 [0.8-4.5]	2.4 [0.6-4.6]	2.2 [0.6 – 4.2]	2.7 [0.6-4.2]
Complete respiratory cycle	3.3 [2.2-8.0]	2.6 [1.5 – 5.5]	4.2 [2.1-7.7]	3.3 [2.1-6.4]	3.2 [1.7-6.0]	3.8 [2.4-6.7]	2.5 [1.2-6.1]	2.7 [0.5-6.8]	2.2 [1.1-6.8]	3.1 [0.8-7.0]	3.0 [0.9 – 7.1]	3.2 [0.8-6.2]
Occupation rate (%)												
Inspiratory phase	19 [9 – 36]	20 [8 – 34]	20 [9 – 36]	25 [13-39]	20 [9 – 42]	22 [8 – 38]	18 [4 – 26]	10 [2 – 32]	13 [3 – 30]	17 [3 – 34]	12 [2 – 37]	20 [2 – 33]
Expiratory phase	32 [16-48]	26 [13 - 41]	36 [18-54]	36 [23 – 49]	31 [17-48]	43 [16 – 55]	29 [10 – 41]	27 [5 – 45]	25 [7 – 46]	30 [7 – 44]	22 [6 – 42]	34 [9 – 60]
Complete respiratory cycle	28 [16 – 40]	25 [10 – 36]	32 [16 – 48]	32 [20 – 42]	29 [16 – 44]	38 [16 – 43]	27 [8 – 35]	24 [4 – 40]	19 [6 – 36]	28 [6 – 41]	20 [6 – 40]	29 [6 – 47]

The values of wheezes obtained in right and left side were pooled.
